# Scrub Typhus as a Cause of Acute Encephalitis Syndrome, Gorakhpur, Uttar Pradesh, India 

**DOI:** 10.3201/eid2308.170025

**Published:** 2017-08

**Authors:** Mahima Mittal, Jeromie Wesley Vivian Thangaraj, Winsley Rose, Valsan Philip Verghese, C.P. Girish Kumar, Mahim Mittal, R. Sabarinathan, Vijay Bondre, Nivedita Gupta, Manoj V. Murhekar

**Affiliations:** BRD Medical College, Gorakhpur, India (Mahima Mittal, Mahim Mittal);; National Institute of Epidemiology, Chennai, India (J.W.V. Thangaraj, C.P. Girish Kumar, R. Sabarinathan, M.V. Murhekar);; Christian Medical College, Vellore, India (W. Rose, V.P. Verghese);; National Institute of Virology, Gorakhpur (V. Bondre);; Indian Council of Medical Research, Delhi, India (N. Gupta)

**Keywords:** scrub typhus, India, Gorakhpur, Uttar Pradesh, acute encephalitis syndrome, meningitis/encephalitis, vector-borne infections

## Abstract

Outbreaks of acute encephalitis syndrome (AES) have been occurring in Gorakhpur Division, Uttar Pradesh, India, for several years. In 2016, we conducted a case–control study. Our findings revealed a high proportion of AES cases with *Orientia tsutsugamushi* IgM and IgG, indicating that scrub typhus is a cause of AES.

Outbreaks of acute encephalitis syndrome (AES) with high case-fatality rates have been occurring in Gorakhpur Division of Uttar Pradesh, India, since 1978 (*1*). AES predominantly affects children <15 years of age (*2*), and its etiology has remained largely unknown (*3**,**4*). Studies focusing on AES in the region have documented that cases with unknown etiology accounted for 41.6% of cases in 2011–2012 (*5*) and 59% in 2013–2014 (*6*). 

Investigations conducted during the 2015 outbreak revealed scrub typhus IgM in >60% of AES cases (*7*). The absence of information about IgM positivity from the general population, and the probability of high background antibody levels in areas to which scrub typhus is endemic, led us to conduct an unmatched case–control study in which we compared IgM and IgG seropositivity for *Orientia tsutsugamushi*, the causative agent of scrub typhus, in AES patients and healthy controls.

We conducted the study during August 17–October 16, 2016. Children <15 years of age with AES admitted to the BRD Medical College in Gorakhpur during the study period were recruited to the study if their parents consented to a blood draw and they had siblings <15 years of age. We defined a case of AES as an acute onset of fever and change in mental status or new onset seizures, excluding febrile seizures (*8*), with cerebrospinal fluid pleocytosis (cell counts >5/mm^3^). Controls were healthy children <15 years of age residing in the home (sibling controls) or village (community controls) of AES case-patients. We interviewed mothers and caretakers for information on case-patients and controls.

We collected 2-mL blood samples from case-patients and controls and tested the samples for *O. tsutsugamushi* IgM and IgG using commercial ELISAs (Scrub Typhus Detect; InBios International Inc., Seattle, WA, USA). We considered an optical density value >0.5 to be a positive result (*9*). We compared IgM and IgG positivity among AES case-patients and their controls by calculating crude and adjusted odds ratios (ORs) with 95% CIs.

We enrolled 46 case-patients and 151 controls (69 sibling and 82 community controls) in the study. The median age was 5 (interquartile range 3–9) years for patients and 7 (interquartile range 4–10) years for controls. The case-patients and controls did not differ by age group or sex (Table). The mothers of 54 (35.8%) of the control children reported a history of fever in the past 6 months.

**Table:** Characteristics of patients with scrub typhus and controls in study of acute encephalitis syndrome, Gorakhpur, Uttar Pradesh, India, 2016

Characteristic	No. (%) patients	No. (%) controls	Odds ratio (95% CI)	p value	Adjusted odds ratio (95% CI)
Age group,y					
<5	24 (52.2)	53 (35.1)	2.0 (0.8–5.2)	0.152	7.4 (1.8–31.0)
6–10	15 (32.6)	67 (44.4)	1.0 (0.4–2.7)	0.987	1.8 (0.5–6.8)
11–15	7 (15.2)	31 (20.5)	1		1
Sex					
M	28 (60.9)	82 (54.3)	1.3 (0.7–2.6)	0.433	
F	18 (39.1)	69 (45.7)	1		
*Orientia tsutsugamushi* IgM					
Positive	29 (63.0)	7 (4.6)	35.1 (13.4–92.3)	0.000	29.9 (9.6–92.9)
Negative	17 (37.0)	144 (95.4)	1		1
*O. tsutsugamushi* IgG					
Positive	38 (82.6)	64 (42.4)	6.5 (2.8–14.8)	0.000	3.8 (1.4–10.9)
Negative	8 (17.4)	87 (57.6)	1		1

Common symptoms among the 46 AES case-patients included seizures (69.6%), altered sensorium (52.2%), and vomiting (37%); physical examinations revealed hepatomegaly (43.4%), cervical or inguinal lymphadenopathy (39.1%), and periorbital edema (54.3%). Cerebrospinal fluid was clear in appearance in 43 of the patients we tested. Cell counts ranged from 5–100/mm^3^ in 41 (95.3%) and were >100/mm^3^ in 2 patients. Protein levels in fluid were <45 mg/dL in 8 patients, 46–100 mg/dL in 15, and >100 mg/dL in 20 patients. All AES case-patients were given intravenous azithromycin; 20 patients also received injected ceftriaxone. Eight patients died.

*O. tsutsugamushi* IgM was detected in 29 (63%) case-patients and IgG in 38 (82.6%). For controls, IgM was detected in 7 (4.6%) and IgG in 64 (42.4%) children. Of the 8 fatal cases, 6 patients had *O. tsutsugamushi* IgM and all had IgG. The distribution of optical density values for IgM and IgG among cases and controls are shown in the Appendix (Technical Appendix). Twenty-eight of the 29 IgM-positive cases and 6 of the 7 controls with IgM seroreactivity were also positive for IgG. Of the 7 controls with IgM seroreactivity, 3 had a history of febrile illness in the past 6 months.

The odds of IgM scrub typhus positivity were 35.1 (95% CI 13.4–92.3) times higher among AES case-patients than among controls; when adjusted for age, the odds were 29.9 (95% CI 9.6–92.9) times higher for case-patients. The odds of IgG positivity were 6.5 (2.8–14.8) times higher among AES case-patients than controls, and when adjusted for age, the odds were 3.8 (95% CI 1.4–10.9) times higher for case-patients. When analyzed separately, AES case-patients had higher odds for IgM positivity compared with sibling (OR 25.1, 95% 6.3–99.8) or community (OR 13.2, 95% 2.27–76.7) controls.

Our study had 1 main limitation: patients and controls were selected from the same village and shared the same environmental risk factors. Despite overmatching that underestimates the strength of association, the odds ratios for *O. tsutsugamushi* IgM and IgG positivity were significant. We concluded that the presence of higher levels of *O. tsutsugamushi* IgM and IgG among AES case-patients than among controls indicates a role for scrub typhus in the etiology of AES in Gorakhpur.

Technical Appendix Distribution of optical density (OD) values for *Orientia tsutsugamushi* IgM among patients with scrub typhus and controls in study of acute encephalitis syndrome, Gorakhpur, Uttar Pradesh, India
