# Supplementary material for: Scrub Typhus as a Cause of Acute Encephalitis Syndrome, Gorakhpur, Uttar Pradesh, India
Source: Emerg Infect Dis. 2017 Aug;23(8):1414–6. doi: 10.3201/eid2308.170025 (PMC5547812; doi:10.3201/eid2308.170025)
Supplement: Technical Appendix — Distribution of optical density (OD) values for Orientia tsutsugamushi IgM among patients with scrub typhus and controls in study of acute encephalitis syndrome, Gorakhpur, Uttar Pradesh, India [file 17-0025-Techapp-s1.pdf]

Article DOI: https://doi.org/10.3201/eid2308.170025

Scrub Typhus as a Cause of Acute Encephalitis Syndrome, Gorakhpur, Uttar Pradesh, India

Technical Appendix

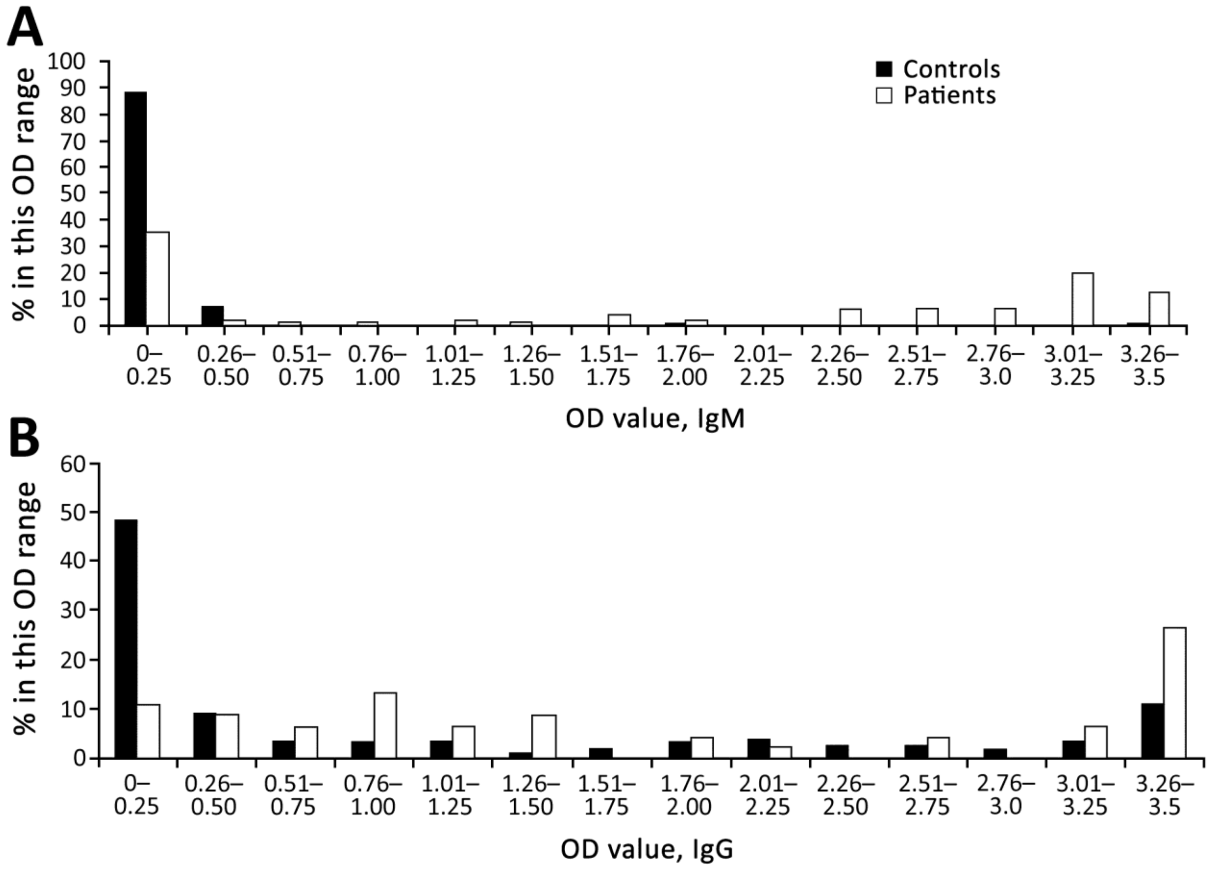

Technical Appendix Figure. Distribution of optical density (OD) values for *Orientia tsutsugamushi* IgM among patients with scrub typhus and controls in study of acute encephalitis syndrome, Gorakhpur, Uttar Pradesh, India
